# CaMKII inhibitors: from research tools to therapeutic agents

**DOI:** 10.3389/fphar.2014.00021

**Published:** 2014-02-20

**Authors:** Patricia Pellicena, Howard Schulman

**Affiliations:** Allosteros Therapeutics, Inc., SunnyvaleCA, USA

**Keywords:** CaMKII, kinase inhibitors, cardiovascular disease, CaMKII inhibitors, AC3-I, KN-93, CaMKIINtide, KN-62

## Abstract

The cardiac field has benefited from the availability of several CaMKII inhibitors serving as research tools to test putative CaMKII pathways associated with cardiovascular physiology and pathophysiology. Successful demonstrations of its critical pathophysiological roles have elevated CaMKII as a key target in heart failure, arrhythmia, and other forms of heart disease. This has caught the attention of the pharmaceutical industry, which is now racing to develop CaMKII inhibitors as safe and effective therapeutic agents. While the first generation of CaMKII inhibitor development is focused on blocking its activity based on ATP binding to its catalytic site, future inhibitors can also target sites affecting its regulation by Ca^2+^/CaM or translocation to some of its protein substrates. The recent availability of crystal structures of the kinase in the autoinhibited and activated state, and of the dodecameric holoenzyme, provides insights into the mechanism of action of existing inhibitors. It is also accelerating the design and development of better pharmacological inhibitors. This review examines the structure of the kinase and suggests possible sites for its inhibition. It also analyzes the uses and limitations of current research tools. Development of new inhibitors will enable preclinical proof of concept tests and clinical development of successful lead compounds, as well as improved research tools to more accurately examine and extend knowledge of the role of CaMKII in cardiac health and disease.

## INTRODUCTION

The search for a multifunctional Ca^2+^-stimulated protein kinase serving to coordinate the actions of Ca^2+^-linked signals, in analogy to the cAMP-dependent protein kinase (PKA) already known to coordinate the actions of cAMP, led to the discovery and characterization of multifunctional Ca^2+^/calmodulin (CaM)-dependent protein kinase II (CaMKII; reviewed in Hudmon and Schulman, 2002; Swulius and Waxham, 2008). Delineation of its functions and relevant substrates was initially complicated by the fact that its activator, Ca^2+^/CaM, regulates many other enzymes. It has therefore been pharmacological inhibitors and genetic ablation or suppression of CaMKII activity that have served to define its functions. This review aims to provide the context for understanding protein kinase inhibition and specifically to describe the types of inhibitors used, their advantages, and their disadvantages. There is now the potential for better inhibitors as therapeutic agents and research tools stemming from industry interest in pursuing CaMKII-based therapeutics, due in no small measure to the cardiovascular scientists in this issue who have identified its critical role in cardiac disease.

## CaMKII STRUCTURE

**FIGURE 1:**
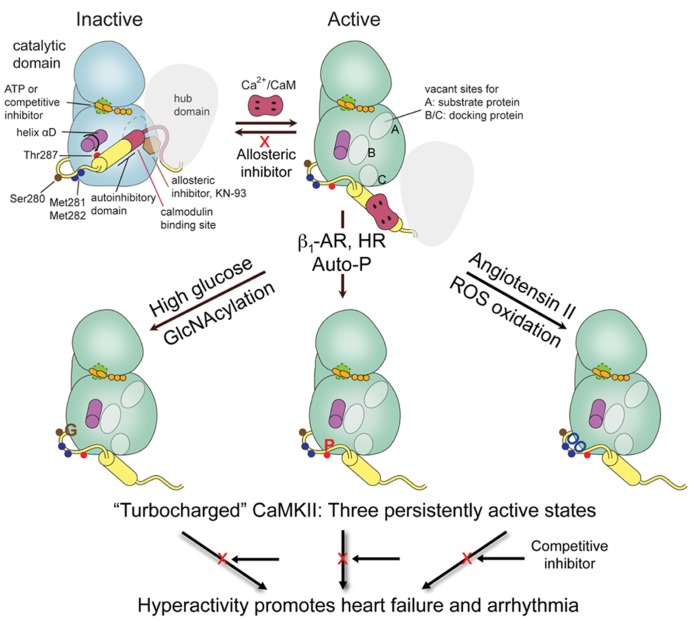
**FIGURE 1. CaMKII domain organization and schematic of activation states.** One subunit of a holoenzyme composed of hub and bi-lobed catalytic domain is shown in the inactive conformation. The autoinhibitory domain or regulatory segment contains the PTM segment, important for regulation by phosphorylation, oxidation, and GlcNAcylation, followed by the CaM binding domain. Binding of Ca^2+^/CaM displaces the autoinhibitory domain leading to rearrangement of the lower or C-lobe of the kinase allowing substrate access to the catalytic cleft and the phosphoacceptor site A, and formation of docking sites B/C that can also be used for substrate specificity. Regulatory modifications at PTM segment by either Ca^2+^/CaM-stimulated autophosphorylation, e.g., by β-adrenergic stimulation or rapid heart rate (HR), oxidation by ROS, e.g., by Angiotensin II signaling, or GlcNAcylation by hyperglycemia disable the autoinhibitory domain to generate a persistently active or “turbocharged” CaMKII leading to cardiac pathophysiology.

Among Ca^2+^/CaM-dependent kinases CaMKII can be best claimed as the multifunctional CaM kinase because it has broad substrate specificity and is ubiquitous, with the γ and δ isoforms present in heart, brain, and other tissues and α and β present at very high levels in brain. Each of these four genes give rise to multiple isoforms, primarily by alternatively spliced sequences (Hudmon and Schulman, 2002; Tombes et al., 2003). The most distinctive feature of CaMKII among protein kinases is that it self-assembles into supramolecular structures of twelve subunits. Each subunit contains an N-terminal catalytic domain followed by a regulatory segment of approximately 40 residues that serves an autoinhibitory function by blocking access to the catalytic site. This domain organization is typical of CaM-regulated protein kinases. The regulatory segment (or autoinhibitory domain) contains most of the elements that are critical for regulation of activity; the posttranslational modification (PTM) segment for regulation by autophosphorylation (Thr287), O-GlcNAC modification (Ser280), and oxidation (Met281/Met282), and the CaM-recognition sequence (**Figure 1**). We will use the amino acid numbering based on the sequence of CaMKIIδ, which are one higher than for the α isoform. Unique to CaMKII is the C-terminal hub or association domain, which is responsible for subunit oligomerization into dodecameric holoenzymes. A flexible linker of variable length connects the regulatory segment to the association domain and it is where most variability resides.

The high resolution crystal structures of the autoinhibited kinase domain and regulatory segment of *C. elegans* CaMKII (Rosenberg et al., 2005) and of all four human isoforms (Rellos et al., 2010) have been elucidated. The structures show a canonical kinase fold with an N-terminal lobe (N-lobe) connected by a “hinge” segment to a C-terminal lobe (C-lobe), where the peptide or protein substrate binding site resides. The ATP-binding site is located at the interface between the two lobes in close proximity to the peptide substrate binding site. In these autoinhibited structures the regulatory segment forms an α-helix of various lengths and folds back onto the kinase domain blocking access to the catalytic site (**Figure 1**). The critical autophosphorylation site, Thr287, is buried at the base of the regulatory segment and inaccessible for phosphorylation. Ca^2+^/CaM binding to the regulatory segment has therefore the dual purpose of first facilitating access to the active site of the kinase by displacing the regulatory segment, and second, to make Thr287 available for phosphorylation *in trans* by a neighboring activated kinase subunit (Hanson et al., 1994). Phosphorylation of Thr287 likely impairs the rebinding of the autoinhibitory domain (Colbran et al., 1989) rendering the kinase “autonomous” of Ca^2+^/CaM and constitutively active until dephosphorylated (reviewed in Hudmon and Schulman, 2002).

The activated state seen in a crystal structure of the kinase domain with the regulatory segment displaced from the kinase domain and bound to Ca^2+^/CaM sheds light on the process of activation by CaM (Rellos et al., 2010). The most notable structural rearrangement is a major reorganization of a helical segment in the C-lobe of the kinase, helix αD (**Figure 1**), impeding the rebinding of the CaM-displaced regulatory segment. The positional shift in helix αD results in the reorientation of Glu97, an important ATP-coordinating residue, leading to a conformation improved for ATP-binding and catalysis (Rosenberg et al., 2005; Rellos et al., 2010). An interesting feature of this “activated” structure is that the regulatory segment adopts an extended conformation and positions Thr287 for capture and autophosphorylation by the active site of a neighboring kinase, as similarly seen in some of the *C. elegans* structures (Chao et al., 2010).

Studying activation states can give insights to additional strategies for inhibitor design (see below). The phosphoacceptor sequence in substrates is positioned at docking site A (previously termed S-site; **Figure 1**; Chao et al., 2010) and has been used in the design of peptide substrates and of “pseudosubstrate” peptides used as inhibitors. An important consequence of helix αD reorientation is the creation of a hydrophobic pocket (first identified and termed docking site B by Chao et al., 2010) that is absent in the autoinhibited form of the kinase. This site anchors hydrophobic residues located five to eight residues N-terminal to the phosphoacceptor site of some substrates for added specificity, and is used for intracellular targeting of the kinase and by peptide inhibitors such as CaMKIINtide (see below). Similarly, an acidic pocket at the base of the C-lobe designated docking site C provides additional interactions for orienting interacting proteins (Chao et al., 2010; **Figure 1**). Docking sites B/C correspond functionally to the region of the molecule referred to as the T-site in previous studies of the autoinhibited state (Hudmon and Schulman, 2002 and references therein). Referring to these as docking sites B and C is now preferred because the site is not just vacated by the regulatory segment during activation but is altered in the process.

The holoenzyme is assembled as two hexameric rings symmetrically stacked one on top of the other with the kinase domains arranged peripherally around a central hub (Woodgett et al., 1983; Kolodziej et al., 2000; Morris and Torok, 2001; Chao et al., 2011). In an isoform lacking the linker domain, the kinase domains nestle between two hub domains with their active sites and regulatory segments completely inaccessible to Ca^2+^/CaM. It is proposed that a dynamic equilibrium governed by the linker length between the kinase and the association domains regulates exposure to CaM-binding sites facilitating the process of holoenzyme activation (Chao et al., 2011).

The PTM segment that enables autonomous activity following autophosphorylation evolved to extend such regulation to reactive oxygen species (ROS) and glucose-linked signaling (**Figure 1**). Increased ROS leads to oxidation of Met 281/282 at the base of the regulatory segment (Erickson et al., 2008). Elevated glucose leads to covalent modification of CaMKII nearby at Ser280 by O-linked N-acetylglucosamine (GlcNAcylation; Erickson et al., 2013). Introduction of bulky groups to the region normally interacting with the surface of the C-lobe is expected to weaken the interaction between the two and, like Thr287 phosphorylation, keep the autoinhibitory domain displaced and the kinase persistently active (**Figure 1**). All three modifications, individually and together, can produce a “turbocharged” kinase with consequences for arrhythmia (Chelu et al., 2009; Purohit et al., 2013), heart failure (Anderson et al., 2011; Luo et al., 2013), asthma (Sanders et al., 2013), and diabetes (Erickson et al., 2013). There may also be additional PTM of CaMKII involving nitrosation of Cys (Gutierrez et al., 2013).

Taken together, the recent accumulation of structural information offers a clearer understanding of CaMKII regulation. These structures not only provide a foundation for the rational design and optimization of CaMKII-specific inhibitors but may also present the opportunity for novel inhibitor-design strategies that extend beyond ATP-binding sites.

## CaMKII INHIBITORS: FROM BENCH TO CLINIC

### KN-93/KN-62

**FIGURE 2:**
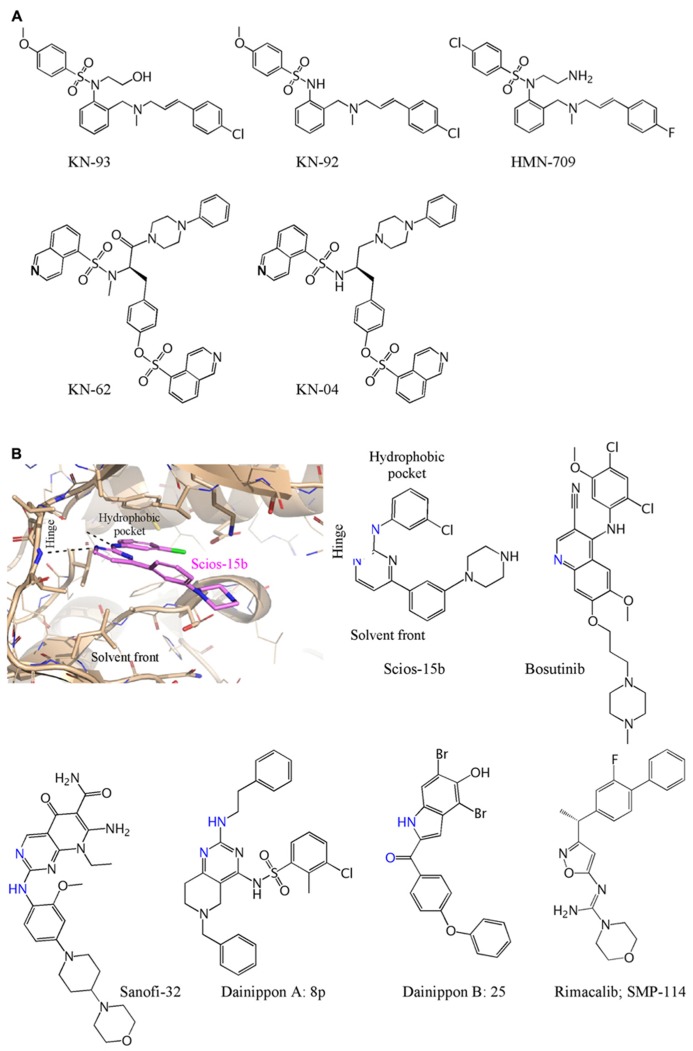
**FIGURE 2. Chemical structures of CaMKII inhibitors. (A)** The ATP non-competitive inhibitors and controls are: KN-93 (Sumi et al., 1991); KN-92 (Tombes et al., 1995); KN-62 and KN-04 (Ishikawa et al., 1990) HMN-709 (Yokokura et al., 1996). **(B)** Computational docking of Compound 15b (Mavunkel et al., 2008) illustrates interaction of an ATP competitive inhibitor viewed from the solvent front and shown docked at the kinase “hinge” and interaction at the hydrophobic pocket. The structure of Compound 15b is shown, with the same orientation, with residues that interact at the hinge in blue based on either a crystal structures (Bosutinib) or based on modeling when a reasonable docked structure could be obtained. The compounds above are: Scios-15b (Mavunkel et al., 2008); Bosutinib (Chao et al., 2011); Sanofi-32 (Beauverger et al., 2012); Dainippon A: 8p (Asano et al., 2010); Dainippon B: 25 (Komiya et al., 2012); Rimacalib/SMP-114 (Westra et al., 2010).

The most widely used inhibitor for study of cellular and *in vivo* functions of CaMKII has been KN-93 (Sumi et al., 1991; **Figure 2A**). It is one of a remarkable number of tool inhibitors developed by Hidaka and his colleagues for PKA, protein kinase C (PKC), CaMKII, and MLCK, many of which became commercially available and widely used. However, these were not on the path to therapeutic use and are therefore not optimized for potency, selectivity, or pharmacokinetics. KN-93 supplanted KN-62 which shares structural elements and mechanism of action (Tokumitsu et al., 1990). Inhibition by both is competitive with Ca^2+^/CaM and not competitive with ATP. KN-62 binds to the holoenzyme and interferes with the ability of Ca^2+^/CaM to activate it, but does not directly bind to CaM, i.e., it is not a CaM antagonist at effective inhibitory concentrations. It is worth noting, however, that a “predecessor” of KN-93 with a very similar structure, HMN-709, is a CaM antagonist (Yokokura et al., 1996; **Figure 2A**). Neither KN-62 nor KN-93 inhibits the activity of autophosphorylated CaMKII, consistent with a block of activation but not of catalysis (Tokumitsu et al., 1990; Sumi et al., 1991). Such inhibition can be classified as “ATP non-competitive” or “allosteric” as binding likely occurs outside the active site. Based on an ischemic stroke model, it has been suggested previously that blocking catalytic activity is the more effective approach because autonomous activity is resistant to allosteric inhibition by KN-93 (Vest et al., 2010). The observed differing efficacies, however, may have been due to differences in inhibitor concentrations at the site of action.

KN-93 (and KN-62) likely blocks the ability of Ca^2+^/CaM to wrap around the CaM-binding segment and free it from the catalytic domain. The displacement of the autoinhibitory regulatory segment can be monitored by appropriate FRET pairs and indeed KN-93 blocks the change in FRET signal induced by Ca^2+^/CaM and the change promoted by either autophosphorylation, oxidation, or GlcNAcylation (Erickson et al., 2011, 2013). KN-93 blocks Ca^2+^/CaM from displacing and “presenting” Thr287 to an active neighboring subunit for phosphorylation (Rich and Schulman, 1998). KN-93 similarly blocks presentation of Ser280 for GlcNAcylation (Erickson et al., 2013). Once activated and autophosphorylated, however, KN-93 does not inhibit the kinase, nor is it likely to inhibit kinase made autonomously active by oxidation or GlcNAcylation.

The initial characterization of KN-93 and KN-62 showed them to be selective for CaMKII relative to PKA, PKC and MLCK (Tokumitsu et al., 1990; Sumi et al., 1991), but they were later shown to inhibit CaMKI and CaMKIV equally well (Mochizuki et al., 1993; Enslen et al., 1994). KN-93 is not very potent, inhibiting CaMKII with an IC50 ~1–4 μM depending on level of CaM and other assay conditions (Sumi et al., 1991; Anderson et al., 1998; Rezazadeh et al., 2006). A recent screen against 234 protein kinases shows that KN-93 is indeed very selective, but its targets now include Fyn, Haspin, Hck, Lck, MLCK, Tec, and TrkA (Gao et al., 2013).

Tool inhibitors are rarely optimized for potency or off-target effects and indeed KN-62 and KN-93, while inhibiting only a few protein kinases, do inhibit many of the ion channels that have been tested. KN-62 and KN-93 block modulation of the L-type Ca^2+^ channel by CaMKII but also have direct effects on the channel (Li et al., 1992; Anderson et al., 1998) so it is important to use their kinase-inactive controls (KN-04 and KN-92). KN-62 and KN-93 block macroscopic voltage-dependent K^+^ current (*K*_v_) in smooth muscle cells at concentrations (0.3–3 μM) used to inhibit CaMKII (Ledoux et al., 1999). KN-92 similarly blocks the channel and is therefore useful in excluding K^+^ channel effects. A detailed analysis demonstrated that KN-93, but not KN-92, blocked members of the Kv1, Kv2, Kv3, Kv4, and Kv7 (hERG) voltage-gated potassium channel families at concentrations that also block CaMKII (Rezazadeh et al., 2006). To be broadly useful as a control, it would be ideal if KN-93 and KN-92 had the same pharmacology other than for CaMKII, but this is unfortunately not the case. For example, KN-92 is not as potent in direct inhibition of L-type Ca^2+^ channels as is KN-93 (Anderson et al., 1998) or as potent in direct inhibition of IP_3_-induced Ca^2+^ release via IP_3_R-1 (Smyth et al., 2002) and thus not every effect blocked by KN-93 and not by KN-92 can be ascribed to CaMKII. Despite these caveats, these inhibitors have been extremely useful as initial evidence for CaMKII action, as illustrated by several examples in **Table 1**.

**Table 1:** CaMKII inhibitors and related compounds.

**Inhibitor type**	**Controls/verification**	**Action of inhibitor**
KN-93/KN-62	KN-92; GFP-AC3-I transgene	KN-93 blocked pacing induced atrial fibrillation in the Ryr2^ R176Q/+^ mouse model (Chelu etal., 2009).
	KN-92; AIP; CaMKIIδ knockout	KN-93, AIP, and knockout block cardiac arrhythmogenesis and sarcoplasmic reticulum Ca^2+^ leak (Sag etal., 2009).
	KN-92	KN-93 and AIP were used to demonstrate that CaMKII is linked to SAN cell bioenergetics, affecting both ATP consumption and ATP generation (Yaniv etal., 2013).
	KN-92; CaMKIIδ knockout	KN-93 blocks increase in GlcNAcylation-dependent Ca^2+^ spark frequency and prevents premature ventricular complexes also seen in diabetes (Erickson etal., 2013).
AC3-I/AIP	GFP-AC3-C	Myocardial GFP-AC3-I transgene blocked maladaptive remodeling following chronic β-adrenergic stimulation or myocardial infarct with GFP-AC3-I (Zhang etal., 2005).
	GFP-AC3-C;KN-93/KN-92	Myocardial GFP-AC3-I transgene in calcineurin hypertrophy model primarily reduced ventricular arrhythmias, improved mechanical function, and decreased mortality with minimal effect on the hypertrophic phenotype (Khoo etal., 2006).
	GFP-AC3-C	AngII promoted AF was blocked by GFP-AC3-I and prevented by knockins with oxidation resistant CaMKII(MM > VV) or RyR2 lacking CaMKII phosphorylation site (RyR2S2814A; Purohit etal., 2013).
	GFP-AC3C; CaMKIIN	Myocardial GFP-AC3-I and blocked increase mortality of diabetic mice after myocardial infarction via reactive oxygen species and confirmed with CaMKII(MM > VV) mice (Luo etal., 2013).
CaMKIIN	Myocardial GFP-AC3-I and -AC3-C	GFP-CaMKIIN (sinoatrial node expressed) blocked isoproterenol-stimulated CaMKII activation and reduced the fight or flight heart rate response to stress or isoproterenol (Wu etal., 2009).
	GFP; AC3-I	GFP-CaMKIIN (sinoatrial node expressed) blocked AngII and ROS activation of CaMKII and cell death contributing to sinus node dysfunction (Swaminathan etal., 2011).
	AC3-I; shRNA; KN-93	HA-CaMKIIN targeted to cytoplasmic membranes acts outside the nucleus to mediate induction of complement factor B following myocardial infarct (Singh etal., 2009).
	CaMKII (Thr287Asp)	mtCaMKIIN (with mitochondrial localization sequence) and palmitoyl-CaMKIIN for membrane localization support a role of mitochondrial CaMKII in ischemia reperfusion injury, MI and neurohumoral injury due to increased inner membrane mitochondrial Ca^2+^ uniporter current (Joiner etal., 2012).

It is a bit surprising, given the channel blocking effects of KN-93, that it has not been as problematic to use it for *in situ* inhibition of CaMKII, when compared to peptide inhibitors below. The interplay between its effect on K^+^ channels and L-type Ca^2+^ channels and inhibition of CaMKII are not well understood and complicate arrhythmia studies. Clearly, modification of K currents, such as in genetic mutations, can lead to arrhythmia, whereas ablation or peptide inhibition of CaMKII are anti-arrhythmic. KN-93 may therefore be anti-arrhythmic despite its K^+^ channel blockade because concurrent inhibition of CaMKII serves as an arrhythmia shield that blocks the pro-arrhythmic consequence of the K^+^ channel inhibition. Alternatively, a significant component of the anti-arrhythmic effect of KN-93 could be to reduce CaMKII activation by inhibiting the L-type Ca^2+^ channel and lowering free Ca^2+^ levels. Ultimately, inhibition of CaMKII with small molecule allosteric or ATP competitive inhibitors can be achieved without a significant K^+^ channel component, something that is harder to achieve with channel blockers used as anti-arrhythmic agents.

### SUBSTRATE-BASED INHIBITORS: AUTOCAMTIDE-3 INHIBITOR (AC3-I)/AUTOCAMTIDE-2 INHIBITOR PROTEINS (AIP)

Identification of the autoinhibitory regulatory segment of CaMKIIα led to development of long inhibitory peptides lacking the CaM binding sequence (residues 273–302) that could be injected into cells, e.g., implicating CaMKII in functions such as long-term potentiation (Payne et al., 1988; Malinow et al., 1989). The N-terminal end of this peptide contains the autophosphorylation site that was the basis for peptides substrates such as autocamtide-2 and autocamtide-3 (Hanson et al., 1989) and substitution of the phosphorylatable Thr to an Ala generated the peptide inhibitors AIP (Ishida et al., 1995) and AC3-I (Braun and Schulman, 1995). They inhibit CaMKII with >100-fold selectivity relative to PKC, PKA and CaMKIV, although their selectivity has not been broadly profiled, e.g., green fluorescent protein (GFP)-AC3-I was found to inhibit cellular actions of protein kinase D1 (PKD1) as well as those of CaMKII (Backs et al., 2009). As exemplified in **Table 1**, peptide inhibitors with internalization sequences for cellular studies or the transgenic expression of the peptides in mice have been critical first steps in delineating cardiovascular functions of CaMKII. Transgenic expression of inhibitor/control pairs can also be used to delineate molecular pathways of gene expression (Singh et al., 2009) and phosphorylation initiated by CaMKII activation (Scholten et al., 2013).

Some caution is warranted in the use of peptide (or small molecule) inhibitors that are often optimistically described as “highly specific inhibitors” when experience or data should suggest otherwise. Useful first generation tool inhibitors are typically developed by academic labs with limited resources, so selectivity is based on a handful of kinases available to the lab rather than on the 50–300 kinases that should be tested. As a minimum, the off-target effects of AC3-I should be checked by use of a control peptide (AC3-C; Patel et al., 1999; Wu et al., 2009). Altered selectivity can also occur when peptides are fused to GFP in order to increase expression and metabolic stability, or modified by lipids or internalization sequences for cell permeation. For example, addition of Ant (Antennapedia) to another peptide inhibitor generated a direct CaM antagonistic sequence (Buard et al., 2010) and myristoylated AIP and AC3-C were shown to have some effects unrelated to CaMKII inhibition (Wu et al., 2009).

### CaMKIIN (CaM-KIIN)

CaMKIIN or CaM-KIIN designates small endogenous proteins that inhibit CaMKII with high affinity that can be applied pharmacologically or genetically. CaMKIIN was discovered by use of a yeast two-hybrid screen whereby the catalytic domain of CaMKIIβ served as bait to clone interacting proteins from a rat neuronal library (Chang et al., 1998, 2001). Two small proteins sharing high homology were identified and termed CaM-KIINβ (79 amino acids) and CaM-KIINα(78 amino acids) to designate them as inhibitors, and often spelled CaMKIIN. The α and β in their names are unrelated to the CaMKII isoform, as either of these inhibits all CaMKII isoforms with IC50 of 50 nM (Chang et al., 2001). The protein has not been detected in heart although a related mRNA was detected (Zhang et al., 2001).

Identification of the core inhibitory domain of CaMKIIN led to generation of a 28 amino acid peptide inhibitor termed CaMKIINtide (Chang et al., 1998) that was subsequently shortened and modified to improve potency (Vest et al., 2007; Coultrap and Bayer, 2011; Gomez-Monterrey et al., 2013). CaMKIIN and CaMKIINtide only bind to the activated conformation of CaMKII, suggesting that they dock to the kinase surface exposed and shaped only after displacement of the autoinhibitory domain during activation. They should therefore inhibit CaMKII activated by bound Ca^2+^/CaM or autonomously active due to autophosphorylation, methionine oxidation, or GlcNAcylation. A 21-residue segment of CaMKIINtide was co-crystallized with CaMKII and shown to dock at the B/C sites using hydrophobic and basic residues at its N-terminal region to support potency and specificity and to extend to the A site where it precludes substrate from binding in a manner similar to protein kinase inhibitor (PKI) binding to PKA (Chao et al., 2010; **Figure 1**).

CaMIINtide has been modified to increase potency (Coultrap and Bayer, 2011; Gomez-Monterrey et al., 2013). In one series of optimizations, a shorter sequence of 21 amino acids (CN21a) was found to retain the potency of CaMKIINtide (Vest et al., 2007). A 19 amino acid sequence was then subjected to Ala scanning substitutions to identify critical residues and subsequent modifications produced a more potent and selective CaMKII inhibitor termed CN19o (Coultrap and Bayer, 2011). CN19o inhibited CaMKIIα with IC50 < 0.4 nM and improved selectivity for tested kinases, showing minimal or no inhibition at 5 μM against CaMKI, CaMKIV, DAPK1, AMPK, PKC, and PKA. A similar study generated a smaller optimized 17 amino acid peptide, CN17β, with IC50 of 30 nM and little inhibition of CaMKI or CaMKIV (Gomez-Monterrey et al., 2013).

CaMKIIN and CaMKIINtides are excellent experimental tools being adopted by the field but their use can result in additional effects by blocking interaction of CaMKII with some anchoring proteins and substrates that share the B/C docking sites (**Figure 1**). Translocation and docking to anchoring proteins aids in fidelity of signal transduction that would be disrupted by CaMKIIN and may generate secondary effects because anchoring proteins often cluster several signaling proteins that might be affected (Colbran, 2004; Schulman, 2004; Tsui et al., 2005). Mutations at the B/C sites block both binding of CN21a and CaMKII translocation/docking to the glutamate NR2B receptor (Leonard et al., 1999; Strack et al., 2000; Bayer and Schulman, 2001; Bayer et al., 2006; Vest et al., 2007) suggesting overlapping binding sites. Application of TatCN21 on neurons inhibits the kinase but also reduces the level of kinase at synaptic sites (Sanhueza et al., 2011), decreases clustering in dendrites, and produces aggregates with polyribosomes (Tao-Cheng et al., 2013). CaMKIINtides also block interaction with densin (Jiao et al., 2011) and Cav2.1 calcium channels (Magupalli et al., 2013) and possibly with β_IV_-spectrin and other proteins (Hund et al., 2010). Finally, the biological function of CaMKIIN is not fully understood and it may directly affect cellular pathways unrelated to CaMKII inhibition or interference with its translocation.

The cardiovascular field has appropriately advanced from the AC3-I/AIP- to CaMKIIN-based inhibitors to delineate *in situ* and *in vivo* functions (**Table 1**). The inhibitor can be directly introduced in a regionally selective manner via locally applied adenoviral constructs, as a transgene targeted to selective tissues, and even directed to distinct intracellular sites with appropriate targeting sequences.

### ERA OF SMALL MOLECULAR THERAPEUTICS

The inhibitory agents and approaches described above have been essential in identifying key roles of CaMKII in health and disease and make a compelling case for targeting CaMKII for several cardiovascular indications, so as one of us asked previously, “where are the drugs?” (Anderson et al., 2006). While it is possible that a CaMKIIN-based inhibitor could be developed as a therapeutic, e.g., for local expression at the sinoatrial node (SAN), new small molecule inhibitors will be needed for treating cardiovascular disease, and these will, in turn, provide better tools for advancing CaMKII research. The period covered since the review above has seen a large increase in the number of protein kinases targeted in oncology, with over 100 hundred in clinical development and many in clinical practice (Cohen and Alessi, 2013). The cardiovascular field, largely because of the greater safety requirements but also because of decades-long investments in ion channel blockers, has been slower in turning to this target class. The message emerging from data published by the CaMKII research field has been heard and now there are several programs targeting CaMKII for cardiovascular indications.

One of the early programs was initiated at Scios, expanded following its acquisition by Johnson and Johnson, and discontinued along with more advanced programs for strategic reasons when Scios was closed. The program did provide some potent ATP competitive inhibitors, along with structure – activity relationships that enables an understanding of how to inhibit CaMKII (Lu et al., 2008; Mavunkel et al., 2008). One of the lead compounds, a pyrimidine (Scios 15b) with IC50 of 9 nM *in vitro* and 320 nM *in situ*, is shown in **Figure 2B**. Bosutinib (and sunitinib) were developed as ATP competitive inhibitors of protein tyrosine kinases but have surprising potency at inhibiting CaMKII and a co-crystal of CaMKII with bosutinib has been published (Chao et al., 2011). Dainippon Sumitomo Pharma has had the most advanced CaMKII program and developed Rimacalib (SMP-114) for treatment of rheumatoid arthritis. It passed the Phase I safety trial, but appears not to have shown efficacy in a 24-week Phase II trial (Tagashira and Fukushima, 2008; Westra et al., 2010). The other compounds are all potent (2–60 nM) inhibitors generated during CaMKII inhibition programs at Sanofi (Beauverger et al., 2012) and Dainippon (Asano et al., 2010; Komiya et al., 2012). Allosteros Therapeutics is developing both ATP-competitive and allosteric or ATP non-competitive inhibitors for cardiovascular and other indications and a CaMKII program at Myogen (with Novartis) was part of an acquisition by Gilead with no publications of structures so far. We are aware of several other early stage CaMKII inhibitor programs and anticipate that both tool inhibitors for academic research as well as new chemical entities for treating cardiovascular disease will arise from several of these programs.

The biochemical properties of some the best characterized CaMKII inhibitors are summarized in **Table 2**.

**Table 2:** Biochemical properties of best characterized CaMKII inhibitors.

Inhibitor	Mechanism of action	Autonomous^a^ kinase inhibition	Off-target effects
KN-93, KN-62	Allosteric, CaM-competitive	No	CaMKI, CaMKIV, ion channels
AC3I/AIP	Peptide substrate-competitive	Yes	PKD-1 in cells
CaMKIIN	Peptide substrate/regulatory domain-competitive	Yes	None identified
Small molecule inhibitors (Scios 15b, SMP-114, Bosutinib)	ATP-competitive	Yes	Inhibit other ser/thr and tyr kinases *in vivo*

a Autonomy is based on Thr287 autophosphorylation but results are likely the same for autonomy generated by regulatory domain methionine oxidation or by GlcNAcylation.

## INHIBITOR DESIGN

CaMKII is now accepted as a key target in cardiovascular disease and the focus is shifting to creation of selective inhibitors that are safe and effective for therapeutic use. The global market for kinase inhibitors is over $30B, mostly targeting protein tyrosine kinases with both biologics and small molecules. Structure-guided drug design and virtual library and fragment screening are likely to benefit from the recent availability of high resolution crystal structures of CaMKII in various conformations. Targeting the ATP-binding site is the most common approach with small molecule inhibitors; however, specificity becomes a challenge because there are over 500 kinases whose active conformation of the site have a similar shape and amino acid composition. Their potency must also be very high in order to compete with millimolar levels of cellular ATP. Successful development of ATP competitive inhibitors for oncology indications has demonstrated that appropriate selectivity is achievable. The first generation of CaMKII therapeutics will likely target the ATP-binding site because of the large body of structural information and medicinal chemistry experience that facilitates the design of relatively selective ATP competitive inhibitors.

The role of CaMKII in cognitive memory and neuronal plasticity that involves its α and β isoforms in brain necessitates development of inhibitors do not block these isoforms in brain, certainly when intended for long-term or chronic use. One approach is gene therapy with viral vectors for expression of peptides or proteins, such as SERCA2a or S100A1 to the heart or specific or very localized regions of heart, such as SAN by intracoronary application or endocardial injection by catheter (Pleger et al., 2013). This type of approach for CaMKII inhibitory proteins, coupled with cardiac-selective promoters, would minimize expression outside the heart and would certainly avoid expression in brain. However, widespread adoption will need to await successful and safe demonstration of exemplary cardiac gene therapy such gene therapy on a larger scale. Gene therapy still holds great promise. For CaMKII inhibition, it promises a lower threshold for success in attaining limiting exposure to heart and avoiding effects in brain and other organs, but it still has a much higher threshold in the development and regulatory path than development of small molecule inhibitors.

For small molecule inhibitors inhibition of α- and β-CaMKII in brain can be minimized by reducing CNS penetration through optimization of their pharmacokinetic properties. There is a large body of literature of physical and structural properties that either promote or limit CNS penetration and typically there are sites on the molecules not involved in target binding that are optimized for solubility, plasma protein binding, and CNS penetration. In addition, while the ATP-binding pockets of the four CaMKII isoforms are similar, it should be possible to further reduce CNS action by perhaps a 10-fold selectivity for δ- over α- and β-CaMKII. The β isoform, in particular, has a slight folding down of the phosphate-binding loop as well as a bulkier amino acid side chain not seen in the other three isoforms making it possible to achieve significant discrimination for this isoform.

A strategy designed to circumvent the high redundancy associated with kinases in the active conformation is to target the more diverse inactive conformation (Huse and Kuriyan, 2002). The anti-cancer drug imatinib (Gleevec®) exhibits a high degree of selectivity for its target Bcr-Abl because, in addition to partially occupying the ATP-binding site, it recognizes a distinctive conformation of the “activation” loop seen only in the inactive state (Schindler et al., 2000; Nagar et al., 2002). It is more difficult to use this strategy for CaMKII because the ATP-binding site is not so different in the active and inactive states. However, because CaMKII is subjected to complex and multilayered mechanisms of regulation, inhibition strategies extending beyond the ATP-binding pocket may be possible.

Allosteric inhibitors, molecules that inhibit enzyme function by binding outside of the active site, show higher selectivity profiles for their targets because such sites are not conserved or even broadly present in the kinome. KN-93 and KN-62 appear to be allosteric inhibitors that may stabilize the interaction between the autoinhibitory regulatory segment and kinase domain and may thereby hinder activation by Ca^2+^/CaM (**Figure 1**). Although the novelty of their unidentified binding sites makes it more challenging to optimize allosteric inhibitors, they offer the advantage of greater selectivity and binding unaffected by high cellular ATP concentrations.

Selectivity would be most easily achieved at unique sites on the kinase. For example, docking sites B/C uncovered in the recent crystal structures may be amenable to medicinal chemistry (Chao et al., 2010; **Figure 1**). It may be possible to block some CaMKII signaling by blocking its targeting to anchoring proteins via the B/C sites as blocking anchoring can decrease phosphorylation even if catalytic activity is not blocked (Tsui et al., 2005). Additionally, it may be possible to achieve target specificity by blocking exposure of the site on substrates or anchoring proteins that interacts with the B/C site. In that case only one or a subset of the multiple CaMKII substrates would be affected. In many cases, however, the phosphorylation site on kinase substrates tends to be exposed, making it difficult to block with a small molecule inhibitor.

The most unique region of CaMKII is the linker region between the catalytic and hub domains (Tombes et al., 2003). Linker length affects the equilibrium between compact and extended holoenzyme conformations and the sensitivity of the kinase to the frequency of Ca^2+^ pulses (Bayer et al., 2002; Chao et al., 2011). Thus, in principle, CaMKII inhibition can be achieved by favoring a more compact holoenzyme and reducing its Ca^2+^ sensitivity. It is likely that the first generation of CaMKII therapeutics will be ATP competitive inhibitors with others following that exploit more unique sites thus enabling fewer off-target effects and safer use for more chronic and less severe indications.

## Conflict of Interest Statement

The authors declare that the research was conducted in the absence of any commercial or financial relationships that could be construed as a potential conflict of interest.

## References

[B1] Anderson M. E. Braun A. P. Wu Y. Lu T. Wu Y. Schulman H. (1998). KN-93, an inhibitor of multifunctional Ca^2+^/calmodulin-dependent protein kinase, decreases early after depolarizations in rabbit heart. *J. Pharmacol. Exp. Ther.* 287 996–1006 9864285

[B2] Anderson M. E. Brown J. H. Bers D. M. (2011). CaMKII in myocardial hypertrophy and heart failure. *J. Mol. Cell Cardiol.* 51 468–473 10.1016/j.yjmcc.2011.01.012 21276796 PMC3158288

[B3] Anderson M. E. Higgins L. S. Schulman H. (2006). Disease mechanisms and emerging therapies: protein kinases and their inhibitors in myocardial disease. *Nat. Clin. Pract. Cardiovasc. Med.* 3 437–445 10.1038/ncpcardio0585 16874356

[B4] Asano S. Komiya M. Koike N. Koga E. Nakatani S. Isobe Y. (2010). 5,6,7,8-Tetrahydropyrido[4,3-d]pyrimidines as novel class of potent and highly selective CaMKII inhibitors. *Bioorg. Med. Chem. Lett.* 20 6696–6698 10.1016/j.bmcl.2010.09.005 20875738

[B5] Backs J. Backs T. Neef S. Kreusser M. M. Lehmann L. H. Patrick D. M. (2009). The δ isoform of CaM kinase II is required for pathological cardiac hypertrophy and remodeling after pressure overload. *Proc. Natl. Acad. Sci. U.S.A.* 106 2342–2347 10.1073/pnas.0813013106 19179290 PMC2650158

[B6] Bayer K. U. De Koninck P. Schulman H. (2002). Alternative splicing modulates the frequency-dependent response of CaMKII to Ca^2+^ oscillations. *EMBO J.* 21 3590–3597 10.1093/emboj/cdf360 12110572 PMC126106

[B7] Bayer K. U. Lebel E. Mcdonald G. L. O’Leary H. Schulman H De Koninck P. (2006). Transition from reversible to persistent binding of CaMKII to postsynaptic sites and NR2B. *J. Neurosci.* 26 1164–1174 10.1523/JNEUROSCI.3116-05.2006 16436603 PMC2890238

[B8] Bayer K. U. Schulman H. (2001). Regulation of signal transduction by protein targeting: the case for CaMKII. *Biochem. Biophys. Res. Commun.* 289 917–923 10.1006/bbrc.2001.6063 11741277

[B9] Beauverger P. Gegis G. Biscarrat S. Duclos O. McCort G. (2012). 5-Oxo-5,8-dihydropyrido[2,3-d]pyrimidine derivatives as CaMKII kinase inhibitors for treating cardiovascular diseases. US Patent, 0 277 220, 2012-11-01.

[B10] Braun A. P. Schulman H. (1995). A non-selective cation current activated via the multifunctional Ca^2+^-calmodulin-dependent protein kinase in human epithelial cells. *J. Physiol. * 488(Pt 1) 37–55 8568664 10.1113/jphysiol.1995.sp020944 PMC1156699

[B11] Buard I. Coultrap S. J. Freund R. K. Lee Y. S. Dell’acqua M. L. Silva A. J. (2010). CaMKII ``autonomy'' is required for initiating but not for maintaining neuronal long-term information storage. *J. Neurosci.* 30 8214–8220 10.1523/JNEUROSCI.1469-10.2010 20554872 PMC2891520

[B12] Chang B. H. Mukherji S. Soderling T. R. (1998). Characterization of a calmodulin kinase II inhibitor protein in brain. *Proc. Natl. Acad. Sci. U.S.A.* 95 10890–10895 10.1073/pnas.95.18.10890 9724800 PMC27991

[B13] Chang B. H. Mukherji S. Soderling T. R. (2001). Calcium/calmodulin-dependent protein kinase II inhibitor protein: localization of isoforms in rat brain. *Neuroscience* 102 767–777 10.1016/S0306-4522(00)00520-0 11182241

[B14] Chao L. H. Pellicena P. Deindl S. Barclay L. A. Schulman H Kuriyan J. (2010). Intersubunit capture of regulatory segments is a component of cooperative CaMKII activation. *Nat. Struct. Mol. Biol.* 17 264–272 10.1038/nsmb.1751 20139983 PMC2855215

[B15] Chao L. H. Stratton M. M. Lee I. H. Rosenberg O. S. Levitz J. Mandell D. J. (2011). A mechanism for tunable autoinhibition in the structure of a human Ca^2+^/calmodulin-dependent kinase II holoenzyme. *Cell* 146 732–745 10.1016/j.cell.2011.07.038 21884935 PMC3184253

[B16] Chelu M. G. Sarma S. Sood S. Wang S. Van Oort R. J. Skapura D. G. (2009). Calmodulin kinase II-mediated sarcoplasmic reticulum Ca^2+^ leak promotes atrial fibrillation in mice. *J. Clin. Invest.* 119 1940–1951 10.1172/JCI37059 19603549 PMC2701862

[B17] Cohen P. Alessi D. R. (2013). Kinase drug discovery–what's next in the field? *ACS Chem. Biol.* 8 96–104 10.1021/cb300610s 23276252 PMC4208300

[B18] Colbran R. J. Smith M. K. Schworer C. M. Fong Y. L. Soderling T. R. (1989). Regulatory domain of calcium/calmodulin-dependent protein kinase II. Mechanism of inhibition and regulation by phosphorylation. *J. Biol. Chem.* 264 4800–4804 2538462

[B19] Colbran R J. (2004). Targeting of calcium/calmodulin-dependent protein kinase II. *Biochem. J.* 378 1–16 10.1042/BJ20031547 14653781 PMC1223945

[B20] Coultrap S. J. Bayer K. U. (2011). Improving a natural CaMKII inhibitor by random and rational design. *PLoS ONE * 6:e25245 10.1371/journal.pone.0025245 PMC3184957 21984908

[B21] Enslen H. Sun P. Brickey D. Soderling S. H. Klamo E. Soderling T. R. (1994). Characterization of Ca^2+^/calmodulin-dependent protein kinase IV. Role in transcriptional regulation. *J. Biol. Chem.* 269 15520–15527 8195196

[B22] Erickson J. R. Joiner M. L. Guan X. Kutschke W. Yang J. Oddis C. V. (2008). A dynamic pathway for calcium-independent activation of CaMKII by methionine oxidation. *Cell* 133 462–474 10.1016/j.cell.2008.02.048 18455987 PMC2435269

[B23] Erickson J. R. Patel R. Ferguson A. Bossuyt J. Bers D. M. (2011). Fluorescence resonance energy transfer-based sensor Camui provides new insight into mechanisms of calcium/calmodulin-dependent protein kinase II activation in intact cardiomyocytes. *Circ. Res.* 109 729–738 10.1161/CIRCRESAHA.111.247148 21835909 PMC3182829

[B24] Erickson J. R. Pereira L. Wang L. Han G. Ferguson A. Dao K. (2013). Diabetic hyperglycaemia activates CaMKII and arrhythmias by O-linked glycosylation. *Nature* 502 372–376 10.1038/nature12537 24077098 PMC3801227

[B25] Gao Y. Davies S. P. Augustin M. Woodward A. Patel U. A. Kovelman R. (2013). A broad activity screen in support of a chemogenomic map for kinase signalling research and drug discovery. *Biochem. J.* 451 313–328 10.1042/BJ20121418 23398362

[B26] Gomez-Monterrey I. Sala M. Rusciano M. R. Monaco S. Maione A. S. Iaccarino G. (2013). Characterization of a selective CaMKII peptide inhibitor. *Eur. J. Med. Chem.* 62 425–434 10.1016/j.ejmech.2012.12.053 23395965

[B27] Gutierrez D. A. Fernandez-Tenorio M. Ogrodnik J. Niggli E. (2013). NO-dependent CaMKII activation during β-adrenergic stimulation of cardiac muscle. *Cardiovasc. Res.* 100 392–401 10.1093/cvr/cvt201 23963842

[B28] Hanson P. I. Kapiloff M. S. Lou L. L. Rosenfeld M. G. Schulman H. (1989). Expression of a multifunctional Ca^2+^/calmodulin-dependent protein kinase and mutational analysis of its autoregulation. *Neuron* 3 59–70 10.1016/0896-6273(89)90115-3 2619995

[B29] Hanson P. I. Meyer T. Stryer L. Schulman H. (1994). Dual role of calmodulin in autophosphorylation of multifunctional CaM kinase may underlie decoding of calcium signals. *Neuron* 12 943–956 10.1016/0896-6273(94)90306-9 8185953

[B30] Hudmon A. Schulman H. (2002). Neuronal Ca^2+^/calmodulin-dependent protein kinase II: the role of structure and autoregulation in cellular function. *Annu. Rev. Biochem.* 71 473–510 10.1146/annurev.biochem.71.110601.135410 12045104

[B31] Hund T. J. Koval O. M. Li J. Wright P. J. Qian L. Snyder J. S. (2010). A β(IV)-spectrin/CaMKII signaling complex is essential for membrane excitability in mice. *J. Clin. Invest.* 120 3508–3519 10.1172/JCI43621 20877009 PMC2947241

[B32] Huse M. Kuriyan J. (2002). The conformational plasticity of protein kinases. *Cell* 109 275–282 10.1016/S0092-8674(02)00741-9 12015977

[B33] Ishida A. Kameshita I. Okuno S. Kitani T. Fujisawa H. (1995). A novel highly specific and potent inhibitor of calmodulin-dependent protein kinase II. *Biochem. Biophys. Res. Commun.* 212 806–812 10.1006/bbrc.1995.2040 7626114

[B34] Ishikawa N. Hashiba Y. Hidaka H. (1990). Effect of a new Ca^2+^-calmodulin-dependent protein kinase II inhibitor on GABA release in cerebrospinal fluid of the rat. *J. Pharmacol. Exp. Ther.* 254 598–602 2384887

[B35] Jiao Y. Jalan-Sakrikar N. Robison A. J. Baucum A. J. II Bass M. A. Colbran R. J. (2011). Characterization of a central Ca^2+^/calmodulin-dependent protein kinase IIalpha/beta binding domain in densin that selectively modulates glutamate receptor subunit phosphorylation. *J. Biol. Chem.* 286 24806–24818 10.1074/jbc.M110.216010 21610080 PMC3137056

[B36] Joiner M. L. Koval O. M. Li J. He B. J. Allamargot C. Gao Z. (2012). CaMKII determines mitochondrial stress responses in heart. *Nature* 491 269–273 10.1038/nature11444 23051746 PMC3471377

[B37] Khoo M. S. Li J. Singh M. V. Yang Y. Kannankeril P. Wu Y. (2006). Death, cardiac dysfunction, and arrhythmias are increased by calmodulin kinase II in calcineurin cardiomyopathy. *Circulation* 114 1352–1359 10.1161/CIRCULATIONAHA.106.644583 16982937

[B38] Kolodziej S. J. Hudmon A. Waxham M. N. Stoops J. K. (2000). Three-dimensional reconstructions of calcium/calmodulin-dependent (CaM) kinase IIα and truncated CaM kinase IIalpha reveal a unique organization for its structural core and functional domains. *J. Biol. Chem.* 275 14354–14359 10.1074/jbc.275.19.14354 10799516

[B39] Komiya M. Asano S. Koike N. Koga E. Igarashi J. Nakatani S. (2012). Synthesis and structure based optimization of 2-(4-phenoxybenzoyl)-5-hydroxyindole as a novel CaMKII inhibitor. *Bioorg. Med. Chem.* 20 6840–6847 10.1016/j.bmc.2012.09.048 23088910

[B40] Ledoux J. Chartier D. Leblanc N. (1999). Inhibitors of calmodulin-dependent protein kinase are nonspecific blockers of voltage-dependent K^+^ channels in vascular myocytes. *J. Pharmacol. Exp. Ther.* 290 1165–74 10454491

[B41] Leonard A. S. Lim I. A. Hemsworth D. E. Horne M. C. Hell J. W. (1999). Calcium/calmodulin-dependent protein kinase II is associated with the N-methyl-\refscd-aspartate receptor. *Proc. Natl. Acad. Sci. U.S.A.* 96 3239–3244 10.1073/pnas.96.6.3239 10077668 PMC15926

[B42] Li G. Hidaka H. Wollheim C. B. (1992). Inhibition of voltage-gated Ca^2+^ channels and insulin secretion in HIT cells by the Ca^2+^/calmodulin-dependent protein kinase II inhibitor KN-62: comparison with antagonists of calmodulin and L-type Ca^2+^ channels. *Mol. Pharmacol.* 42 489–488 1328847

[B43] Lu Q. Chen Z. Perumattam J. Wang D. X. Liang W. Xu Y. J. (2008). Aryl-indolyl maleimides as inhibitors of CaMKIIδ. Part 3: importance of the indole orientation. *Bioorg. Med. Chem. Lett*. 18 2399–2403 10.1016/j.bmcl.2008.02.057 18337095

[B44] Luo M. Guan X. Luczak E. D. Lang D. Kutschke W. Gao Z. (2013). Diabetes increases mortality after myocardial infarction by oxidizing CaMKII. *J. Clin. Invest.* 123 1262–1274 10.1172/JCI65268 23426181 PMC3673230

[B45] Magupalli V. G. Mochida S. Yan J. Jiang X. Westenbroek R. E. Nairn A. C. (2013). Ca^2+^-independent activation of Ca^2+^/calmodulin-dependent protein kinase II bound to the C-terminal domain of CaV2.1 calcium channels. *J. Biol. Chem. * 288 4637–4648 10.1074/jbc.M112.369058 23255606 PMC3576069

[B46] Malinow R. Schulman H. Tsien R. W. (1989). Inhibition of postsynaptic PKC or CaMKII blocks induction but not expression of LTP. *Science* 245 862–866 10.1126/science.2549638 2549638

[B47] Mavunkel B. Xu Y. J. Goyal B. Lim D. Lu Q. Chen Z. (2008). Pyrimidine-based inhibitors of CaMKIId. *Bioorg. Med. Chem. Lett.* 18 2404–2408 10.1016/j.bmcl.2008.02.056 18334293

[B48] Mochizuki H. Ito T. Hidaka H. (1993). Purification and characterization of Ca^2+^/calmodulin-dependent protein kinase V from rat cerebrum. *J. Biol. Chem.* 268 9143–9147 8386178

[B49] Morris E. P. Torok K. (2001). Oligomeric structure of alpha-calmodulin-dependent protein kinase II. *J. Mol. Biol.* 308 1–8 10.1006/jmbi.2001.4584 11302701

[B50] Nagar B. Bornmann W. G. Pellicena P. Schindler T. Veach D. R. Miller W. T. (2002). Crystal structures of the kinase domain of c-Abl in complex with the small molecule inhibitors PD173955 and imatinib (STI-571). *Cancer Res.* 62 4236–4243 12154025

[B51] Patel R. Holt M. Philipova R. Moss S. Schulman H. Hidaka H. (1999). Calcium/calmodulin-dependent phosphorylation and activation of human Cdc25-C at the G2/M phase transition in HeLa cells. *J. Biol. Chem.* 274 7958–7968 10.1074/jbc.274.12.7958 10075693

[B52] Payne M. E. Fong Y. L. Ono T. Colbran R. J. Kemp B. E. Soderling T. R. (1988). Calcium/calmodulin-dependent protein kinase II. Characterization of distinct calmodulin binding and inhibitory domains. *J. Biol. Chem.* 263 7190–7195 2835367

[B53] Pleger S. T. Brinks H. Ritterhoff J. Raake P. Koch W. J. Katus H. A. (2013). Heart failure gene therapy: the path to clinical practice. *Circ. Res.* 113 792–809 10.1161/CIRCRESAHA.113.300269 23989720 PMC11848682

[B54] Purohit A. Rokita A. G. Guan X. Chen B. Koval O. M. Voigt N. (2013). Oxidized Ca^2+^/calmodulin-dependent protein kinase II triggers atrial fibrillation. *Circulation* 128 1748–1757 10.1161/CIRCULATIONAHA.113.003313 24030498 PMC3876034

[B55] Rellos P. Pike A. C. W. Niesen F. H. Salah E. Lee W. H. Von Delft F. (2010). Structure of the CaMKIIδ/calmodulin complex reveals the molecular mechanism of CaMKII kinase activation. *PLoS Biol. * 8:e1000426 10.1371/journal.pbio.1000426 PMC2910593 20668654

[B56] Rezazadeh S. Claydon T. W. Fedida D. (2006). KN-93 (2-[N-(2-hydroxyethyl)]-N-(4-methoxybenzenesulfonyl)]amino-N-(4-chlorocinn amyl)-N-methylbenzylamine), a calcium/calmodulin-dependent protein kinase II inhibitor, is a direct extracellular blocker of voltage-gated potassium channels. *J. Pharmacol. Exp. Ther.* 317 292–299 10.1124/jpet.105.097618 16368898

[B57] Rich R. C. Schulman H. (1998). Substrate-directed function of calmodulin in autophosphorylation of Ca^2+^/calmodulin-dependent protein kinase II. *J. Biol. Chem.* 273 28424–28429 10.1074/jbc.273.43.28424 9774470

[B58] Rosenberg O. S. Deindl S. Sung R. J. Nairn A. C. Kuriyan J. (2005). Structure of the autoinhibited kinase domain of CaMKII and SAXS analysis of the holoenzyme. *Cell* 123 849–860 10.1016/j.cell.2005.10.029 16325579

[B59] Sag C. M. Wadsack D. P. Khabbazzadeh S. Abesser M. Grefe C. Neumann K. (2009). Calcium/calmodulin-dependent protein kinase II contributes to cardiac arrhythmogenesis in heart failure. *Circ. Heart Fail.* 2 664–675 10.1161/CIRCHEARTFAILURE.109.865279 19919992 PMC2835502

[B60] Sanders P. N. Koval O. M. Jaffer O. A. Prasad A. M. Businga T. R. Scott J. A. (2013). CaMKII is essential for the proasthmatic effects of oxidation. *Sci. Transl. Med.* 5 195ra197 10.1126/scitranslmed.3006135 PMC4331168 23884469

[B61] Sanhueza M. Fernandez-Villalobos G. Stein I. S. Kasumova G. Zhang P. Bayer K. U. (2011). Role of the CaMKII/NMDA receptor complex in the maintenance of synaptic strength. *J. Neurosci.* 31 9170–9178 10.1523/JNEUROSCI.1250-11.2011 21697368 PMC3138556

[B62] Schindler T. Bornmann W. Pellicena P. Miller W. T. Clarkson B. Kuriyan J. (2000). Structural mechanism for STI-571 inhibition of abelson tyrosine kinase. *Science* 289 1938–1942 10.1126/science.289.5486.1938 10988075

[B63] Scholten A. Preisinger C. Corradini E. Bourgonje V. J. Hennrich M. L. Van Veen T. A. (2013). Phosphoproteomics study based on in vivo inhibition reveals sites of calmodulin-dependent protein kinase II regulation in the heart. *J. Am. Heart Assoc.* 2:e000318 10.1161/JAHA.113.000318 PMC3828808 23926118

[B64] Schulman H. (2004). Activity-dependent regulation of calcium/calmodulin-dependent protein kinase II localization. *J. Neurosci.* 24 8399–8403 10.1523/JNEUROSCI.3606-04.2004 15456811 PMC6729891

[B65] Singh M. V. Kapoun A. Higgins L. Kutschke W. Thurman J. M. Zhang R. (2009). Ca^2+^/calmodulin-dependent kinase II triggers cell membrane injury by inducing complement factor B gene expression in the mouse heart. *J. Clin. Invest.* 119 986–996 10.1172/JCI35814 19273909 PMC2662543

[B66] Smyth J. T. Abbott A. L. Lee B. Sienaert I. Kasri N. N. De Smedt H. (2002). Inhibition of the inositol trisphosphate receptor of mouse eggs and A7r5 cells by KN-93 via a mechanism unrelated to Ca^2+^/calmodulin-dependent protein kinase II antagonism. *J. Biol. Chem.* 277 35061–35070 10.1074/jbc.M202928200 12121980

[B67] Strack S. Mcneill R. B. Colbran R. J. (2000). Mechanism and regulation of calcium/calmodulin-dependent protein kinase II targeting to the NR2B subunit of the N-methyl-\refscd-aspartate receptor. *J. Biol. Chem.* 275 23798–23806 10.1074/jbc.M001471200 10764765

[B68] Sumi M. Kiuchi K. Ishikawa T. Ishii A. Hagiwara M. Nagatsu T. (1991). The newly synthesized selective Ca^2+^/calmodulin dependent protein kinase II inhibitor KN-93 reduces dopamine contents in PC12h cells. *Biochem. Biophys. Res. Commun.* 181 968–975 10.1016/0006-291X(91)92031-E 1662507

[B69] Swaminathan P. D. Purohit A. Soni S. Voigt N. Singh M. V. Glukhov A. V. (2011). Oxidized CaMKII causes cardiac sinus node dysfunction in mice. *J. Clin. Invest.* 121 3277–3288 10.1172/JCI57833 21785215 PMC3223923

[B70] Swulius M. T. Waxham M. N. (2008). Ca^2+^/calmodulin-dependent protein kinases. *Cell Mol. Life Sci.* 65 2637–2657 10.1007/s00018-008-8086-2 18463790 PMC3617042

[B71] Tagashira S. Fukushima A. (2008). Combination drug for treating autoimmune disease. US Patent, 0 255 121 2008-10-16.

[B72] Tao-Cheng J. H. Yang Y. Bayer K. U. Reese T. S. Dosemeci A. (2013). Effects of CaMKII inhibitor tatCN21 on activity-dependent redistribution of CaMKII in hippocampal neurons. *Neuroscience* 244 188–196 10.1016/j.neuroscience.2013.03.063 23583761 PMC3673545

[B73] Tokumitsu H. Chijiwa T. Hagiwara M. Mizutani A. Terasawa M. Hidaka H. (1990). KN-62, 1-[N,O-bis(5-isoquinolinesulfonyl)-N-methyl-\refscl-tyrosyl]-4-phenylpiperazine, a specific inhibitor of Ca^2+^/calmodulin-dependent protein kinase II. *J. Biol. Chem.* 265 4315–4320 2155222

[B74] Tombes R. M. Faison M. O. Turbeville J. M. (2003). Organization and evolution of multifunctional Ca^2+^/CaM-dependent protein kinase genes. *Gene* 322 17–31 10.1016/j.gene.2003.08.023 14644494

[B75] Tombes R. M. Grant S. Westin E. H. Krystal G. (1995). G1 cell cycle arrest and apoptosis are induced in NIH 3T3 cells by KN-93, an inhibitor of CaMK-II (the multifunctional Ca^2+^/CaM kinase). *Cell Growth Differ.* 6 1063–1070 8519682

[B76] Tsui J. Inagaki M. Schulman H. (2005). Calcium/calmodulin-dependent protein kinase II (CaMKII) localization acts in concert with substrate targeting to create spatial restriction for phosphorylation. *J. Biol. Chem.* 280 9210–9216 10.1074/jbc.M407653200 15582994

[B77] Vest R. S. Davies K. D. O’leary H. Port J. D. Bayer K. U. (2007). Dual mechanism of a natural CaMKII inhibitor. *Mol. Biol. Cell* 18 5024–5033 10.1091/mbc.E07-02-0185 17942605 PMC2096578

[B78] Vest R. S. O’leary H. Coultrap S. J. Kindy M. S. Bayer K. U. (2010). Effective post-insult neuroprotection by a novel CaMKII inhibitor. *J. Biol. Chem.* 285 20675–20682 10.1074/jbc.M109.088617 20424167 PMC2898334

[B79] Westra J. Brouwer E. Van Roosmalen I. A. M. Doornbos-Van Der Meer B. Van Leeuwen M. A. Posthumus M. D. (2010). Expression and regulation of HIF-1alpha in macrophages under inflammatory conditions; significant reduction of VEGF by CaMKII inhibitor. *BMC Musculoskelet. Disord. * 11:61 10.1186/1471-2474-11-61. PMC2851671 20353560

[B80] Woodgett J. R. Davison M. T. Cohen P. (1983). The calmodulin-dependent glycogen synthase kinase from rabbit skeletal muscle. Purification, subunit structure and substrate specificity. *Eur. J. Biochem.* 136 481–487 10.1111/j.1432-1033.1983.tb07766.x 6315430

[B81] Wu Y. Gao Z. Chen B. Koval O. M. Singh M. V. Guan X. (2009). Calmodulin kinase II is required for fight or flight sinoatrial node physiology. *Proc. Natl. Acad. Sci. U.S.A.* 106 5972–5977 10.1073/pnas.0806422106 19276108 PMC2667018

[B82] Yaniv Y. Spurgeon H. A. Ziman B. D. Lakatta E. G. (2013). Ca^2+^/calmodulin-dependent protein kinase II (CaMKII) activity and sinoatrial nodal pacemaker cell energetics. *PLoS ONE * 8:e57079 10.1371/journal.pone.0057079 PMC3581576 23459256

[B83] Yokokura H. Okada Y. Terada O. Hidaka H. (1996). HMN-709, a chlorobenzenesulfonamide derivative, is a new membrane-permeable calmodulin antagonist. *Jpn. J. Pharmacol.* 72 127–135 10.1254/jjp.72.127 8912914

[B84] Zhang J. Li N. Yu J. Zhang W. Cao X. (2001). Molecular cloning and characterization of a novel calcium/calmodulin-dependent protein kinase II inhibitor from human dendritic cells. *Biochem. Biophys. Res. Commun.* 285 229–234 10.1006/bbrc.2001.5175 11444830

[B85] Zhang R. Khoo M. S. Wu Y. Yang Y. Grueter C. E. Ni G. (2005). Calmodulin kinase II inhibition protects against structural heart disease. *Nat. Med*. 11 409–417 10.1038/nm1215 15793582

